# Locomotor rhythm and pattern generating networks of the human lumbar spinal cord: an electrophysiological and computer modeling study

**DOI:** 10.1186/1471-2202-14-S1-P274

**Published:** 2013-07-08

**Authors:** Simon M Danner, Frank Rattay, Ursula S Hofstoetter, Milan R Dimitrijevic, Karen Minassian

**Affiliations:** 1 Institute for Analysis and Scientific Computing, Vienna University of Technology, Vienna, Austria; 2 Center for Medical Physics and Biomedical Engineering, Medical University of Vienna, Vienna, Austria; 3 Baylor College of Medicine, Houston, TX, USA

## 

The concept of the neural control of human locomotion has undergone changes in the past decades. In spite of the encephalization and the erect, bipedal mode of walking, independent observations imply that unperturbed locomotor patterns can be generated by similar spinal neural circuits as in other vertebrates [1,2]. However, little is known about the organization of these rhythm and pattern generating networks in humans. It has been shown that the human lumbar spinal cord isolated from supraspinal control due to traumatic spinal cord injury (SCI) can generate rhythmic, locomotor-like activity in response to sustained epidural spinal cord stimulation of certain frequencies [1]. In the supine position, in which the subjects were tested, afferent feedback is minimized. The rhythmic activities consist of a series of stimulus time-related rhythmically modulated posterior-root muscle (PRM) reflexes, each initiated in posterior root afferents and electromyographically recorded as compound muscle action potentials (CMAPs) [3]. The relation between individual stimuli and responses, as well as their characteristics, allow for the identification of mechanisms beyond the information gained from the overall electromyographic (EMG) patterns.

Here, EMG activities of quadriceps, hamstrings, tibialis anterior and triceps surae, bilaterally in response to epidural stimulation at 20 Hz-40 Hz were analyzed in 10 individuals with motor complete posttraumatic SCI. Forty segments (duration: 10 s) of rhythmical activities found in all four-muscle groups of one lower limb at the same time were identified in 7 subjects. Phases of bursting and suppressed activities were identified. Latencies of PRM reflexes were calculated. Furthermore, a computational network model of neurons with Hodgkin-Huxley-like membrane dynamics was developed to test whether hypothesized rhythm and pattern generating networks would reproduce the recorded data. A core rhythm-generating network model [4] was extended by adding conduction delays, presynaptic inhibition and dis-inhibition of parallel central pathways.

In all 10-s segments, rhythmical activities of all muscle groups had the same cycle frequency. In-between muscles, rhythmic activity occurred either synchronous or alternating, within phases resembling flexion or extension. No other phase-relations were observed. PRM reflexes constituting bursts during the extension phases had monosynaptic latencies. These responses were suppressed during flexion and were replaced by delayed, oligosynaptic PRM reflexes in quadriceps, tibialis anterior and triceps surae. Computer simulation confirmed that a network model with a half-center organization, phasic modulation of the monosynaptic reflex gain, presynaptic control of primary afferents together with the selection of alternative interneuronal pathways (within the flexor half-center) reliably reproduce the electrophysiological findings.

The electrophysiological data as well as the computer simulations give insight into the organization of the human spinal rhythm and pattern generating networks and reveal common control characteristics with the central pattern generators for locomotion described in animal experimental work.
